# Simultaneous amplification of multiple immunofluorescence signals via cyclic staining of target molecules using mutually cross-adsorbed antibodies

**DOI:** 10.1038/s41598-022-12808-y

**Published:** 2022-05-24

**Authors:** Haemin Yeon, Yehlin Cho, Junyoung Seo, Yeonbo Sim, Jae-Byum Chang

**Affiliations:** 1 Department of Materials Science and Engineering, Korea Advanced Institute of Science and Technology (KAIST), Daejeon, 34141 Republic of Korea; 2 Department of Materials Science and Engineering, Massachusetts Institute of Technology (MIT), Cambridge, MA 02139 USA; 3 Department of Biomedical Engineering, Sungkyunkwan University, Suwon, 16419 Republic of Korea

**Keywords:** Biochemistry, Biological techniques, Molecular biology

## Abstract

Amplification of immunofluorescence (IF) signals is becoming increasingly critical in cancer research and neuroscience. Recently, we put forward a new signal amplification technique, which we termed fluorescent signal amplification via cyclic staining of target molecules (FRACTAL). FRACTAL amplifies IF signals by repeatedly labeling target proteins with a pair of secondary antibodies that bind to each other. However, simultaneous amplification of multiple IF signals via FRACTAL has not yet been demonstrated because of cross-reactivity between the secondary antibodies. In this study, we show that mutual cross-adsorption between antibodies can eliminate all forms of cross-reactions between them, enabling simultaneous amplification of multiple IF signals. First, we show that a typical cross-adsorption process—in which an antibody binds to proteins with potential cross-reactivity with the antibody—cannot eliminate cross-reactions between antibodies in FRACTAL. Next, we show that all secondary antibodies used in FRACTAL need to be mutually cross-adsorbed to eliminate all forms of cross-reactivity, and then we demonstrate simultaneous amplification of multiple IF signals using these antibodies. Finally, we show that multiplexed FRACTAL can be applied to expansion microscopy to achieve higher fluorescence intensities after expansion. Multiplexed FRACTAL is a highly versatile tool for standard laboratories, as it amplifies multiple IF signals without the need for custom antibodies.

## Introduction

Amplification of multiple immunofluorescent signals is particularly useful in neuroscience and cancer biology^1^. For example, in cancer biology, it is sometimes difficult to detect proteins pertinent to a prognosis using immunofluorescence imaging because of the low expression level of these proteins^2^. In neuroscience, imaging protein expression profiles over a large volume, e.g., the entire brain, is becoming critical^3^; and the total imaging time required for such large-volume imaging can be prohibitively long. However, the total imaging time can be reduced using signal amplification^4^. Furthermore, in both cancer biology and neuroscience, multiplexed imaging of more than three or four proteins is required to identify the overall spatiotemporal distribution of the proteins and the relations between target proteins^5^. Therefore, a signal amplification technique that can simultaneously amplify multiple fluorescence signals is needed.

Consequently, a number of signal amplification methods have been developed, including tyramide signal amplification (TSA)^6^, avidin–biotin complex (ABC) formation^7^, and oligonucleotide-based techniques^8^. In TSA, the target proteins are first labeled with horseradish peroxidase (HRP)-conjugated antibodies. The HRP then induces the deposition of fluorophore-labeled tyramides around the target proteins, which amplifies the fluorescence signal^6^. In ABC, the target proteins are labeled with avidin-labeled antibodies. Multiple fluorophore-conjugated biotins then bind to the avidin and the fluorescence signals are amplified^7^. TSA and ABC can amplify fluorescence signals more than eighty and sixfold, respectively^9,10^. However, these two techniques rely on a single chemistry, and for multiplexed signal amplification, the signal amplification process needs to be repeated multiple times for each signal^7,11^. Conversely, oligonucleotide-based signal amplification can simultaneously amplify multiple fluorescence signals with the target proteins labeled with oligonucleotide-labeled antibodies and the hybridization of fluorophore-conjugated complementary oligonucleotides^8^. However, this technique requires oligonucleotide-conjugated antibodies, which are difficult to prepare manually in most biology laboratories.

**Figure 1:**
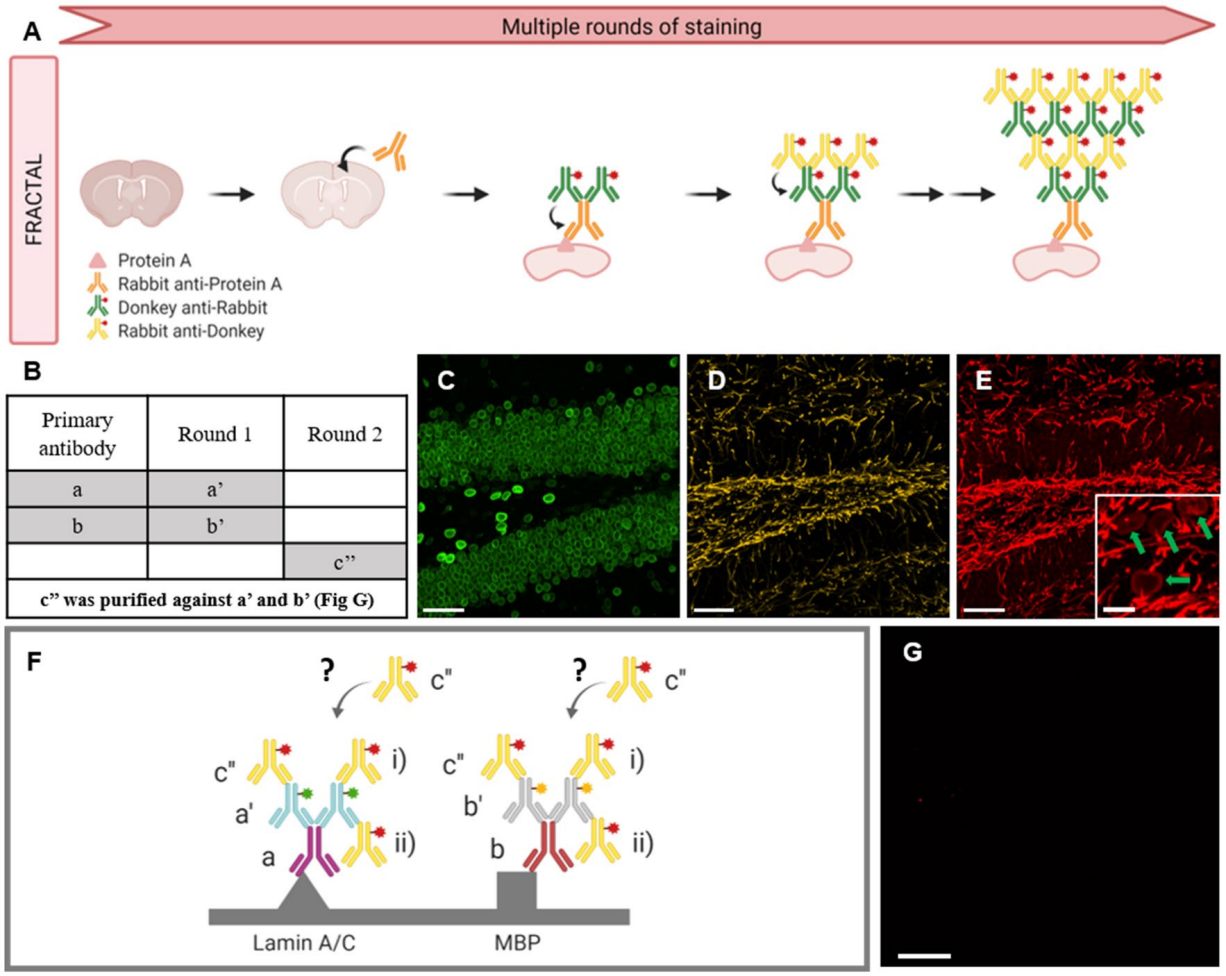
Generic FRACTAL and purification processes. (**A**) Schematics of the generic FRACTAL process. (**B**) Purification table of the secondary antibodies used, illustrated in (**G**). (**C**–**E**) Cross-reactivity test of unpurified and purified CF633-conjugated rb anti-dk secondary antibody in a mouse brain slice: (**C**) 488-nm channel showing lamin A/C. (**D**) 568-nm channel showing MBP. (**E**) 633-nm channel, using unpurified CF633-conjugated rb anti-dk secondary antibody; overlaps with the 488-nm and 568-nm channels, and the inset overlaps with the lamin A/C channel (See green arrows) (**F**) Possible schematics of the cross-reaction shown in (**E**). (**G**) 633-nm channel, using purified CF633-conjugated rb anti-dk secondary antibody; no signal was observed. For (**C**,**D**,**E**), and (**G**), the scale bar is 30 $\mu$m. For the inset in (**E**), the scale bar is 7.5 $\mu$m.

To solve these problems, we previously developed a new signal amplification technique, termed fluorescent signal amplification via cyclic staining of target molecules (FRACTAL)^12^. In FRACTAL, target proteins are labeled with primary antibodies and fluorophore-conjugated first secondary antibodies. The first secondary antibodies are then labeled with second secondary antibodies (Fig. 1A). By repeating the staining of specimens with first and second secondary antibodies, the fluorescence signals of each target protein can be amplified. In FRACTAL, a pair of secondary antibodies is used for each fluorescence signal. On the other hand, simultaneously amplifying three fluorescence signals using FRACTAL requires the use of six secondary antibodies produced from different host species. However, simultaneous multiplexed signal amplification through FRACTAL has not yet been demonstrated because of the cross-reactivity between secondary antibodies.

In this study, we demonstrate simultaneous multiplexed signal amplification via FRACTAL by eliminating the cross-reactivity between six secondary antibodies using a mutual cross-adsorption process. We first show that there is a high level of cross-reactivity between secondary antibodies when signal amplification is performed without any cross-adsorption. Then we show that such cross-reactivity can be eliminated via single-step mutual cross-adsorption between secondary antibodies, and we demonstrate multiplexed signal amplification using these cross-adsorbed secondary antibodies. Here, the fluorescence signals of three different types of proteins; lamin A/C, myelin basic protein (MBP), and glial fibrillary acidic protein (GFAP) were simultaneously amplified. These three proteins were chosen to demonstrate the ability of FRACTAL to enhance the signals of a variety of proteins. Lamin A/C is a cellular organelle marker expressed in the nuclear membrane^13^. MBP is a subcellular neuronal structural marker found in the myelinated axons of neurons^14^. GFAP is a cell-type marker expressed only by astrocytes^15^. FRACTAL was shown to work for cellular organelles, subcellular neuronal structures, and cell type markers. These three protein types play a vital role in brain function and are a common subject in biological research. By choosing these proteins, we tried to validate that the fluorescence signals of various proteins can be amplified simultaneously. Finally, we combine multiplexed FRACTAL with protein-retention expansion microscopy (proExM) and demonstrate super-resolution imaging of three proteins in a mouse brain using signal amplification^16^. This multiplexed FRACTAL provides an effective tools for simultaneous multiplexed signal amplification imaging of proteins of interest.

## Results

### Optimization of purification protocol for eliminating the crosstalk between secondary antibodies

First, we applied the FRACTAL process to multiple target proteins using multiple secondary antibody pairs without any cross-adsorption processes. We stained a mouse brain slice with mouse (ms) anti-lamin A/C (denoted by a in Fig. 1B) and chicken (ck) anti- MBP (denoted by b in Fig. 1B) antibodies. Then, we applied two first secondary antibodies: CF488-conjugated rat (rt) anti-ms (denoted by a’ in Fig. 1B) and CF568-conjugated goat (gt) anti-ck (denoted by b’ in Fig. 1B). Subsequently, we applied a CF633-conjugated rabbit (rb) anti-donkey (dk) secondary antibody (denoted by c’’ in Fig. 1B), which does not bind to any antibodies in the specimens. As expected, only the lamin A/C structures were imaged in the 488-nm channel while MBP structures were imaged in the 560-nm channel (Fig. 1D,E). No fluorescence signal should be detected in the 633-nm channel, but both the lamin A/C and MBP structures were imaged (Fig. 1F). Such cross-reactivity occurs if the second secondary antibody —an CF633-conjugated rb anti-dk antibody— binds to the first secondary antibodies (i in Fig. 1F), or if this antibody is captured by the free-antigen binding sites of the two first secondary antibodies (ii in Fig. 1F). In a separate experiment, we found crosstalk between other antibodies, which were a cross-reaction of a ck anti-gt antibody with rt anti-ms and dk anti-rb antibodies (Fig. S1A to S1C).

To eliminate such cross-reactivity, we performed cross-adsorption of secondary antibodies using a commercial affinity purification column designed to collect antibodies with a strong affinity for a specific target (Fig. S2A). The objective of our experiment was to collect antibodies with no affinity for specific targets (Fig. S2B). Therefore, we functionalized N-hydroxysuccinimide (NHS)-activated agarose beads in the columns with an rt anti-ms antibody (denoted by a’ in Fig. 1C) and/or a gt anti-ck antibody (denoted by b’ in Fig. 1C) and applied an rb anti-dk antibody (denoted by c’’ in Fig. 1C) antibody to the column, and then we collected the flow through (Fig. S2B). When applying an rb anti-dk antibody to the column, we used a CF 633-conjugated antibody instead of an unconjugated antibody.

**Figure 2:**
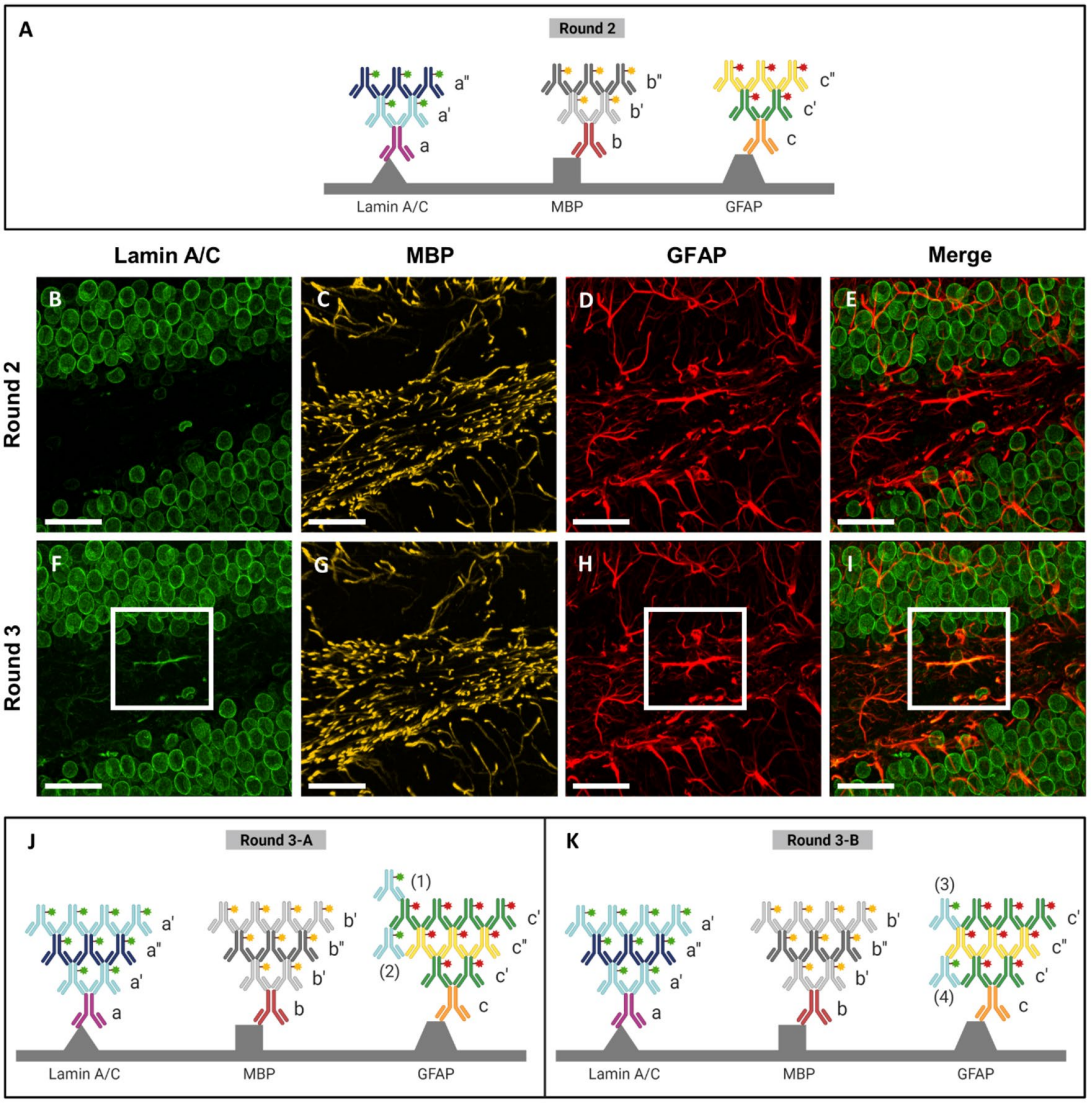
Cross-reaction during Round 3 of staining. (**A**) Schematics of the antibody layers in Round 2. (**B**)–(**E**): Round 2 images, with no cross-reaction observed between lamin A/C and GFAP channel. (**B**) lamin A/C, Round 2. (**C**) MBP, Round 2. (**D**) GFAP, Round 2. (**E**) Merge of Fig. 1B,D. (**F**–**I**): Round 3 images, with cross-reaction observed between lamin A/C and GFAP channel. (**F**) lamin A/C, Round 3. (**G**) MBP, Round 3. (**H**) GFAP, Round 3. (**I**) Merge of Fig. 1F,H. (**J**) Schematics of the first potential cross-reaction during Round 3. (**K**) Schematics of the second potential cross-reaction in Round 3. For (**B**–**I**), the scale bar is 30 $\mu$m.

In performing cross-adsorption of the CF633-conjugated rb anti-dk against the two first secondary antibodies, we investigated two protocols: serial cross-adsorption and simultaneous cross-adsorption (Fig. S3). With serial cross-adsorption, the CF633-conjugated rb anti-dk was cross-adsorbed against one of the two first secondary antibodies and then the flow through was cross-adsorbed against the other first secondary antibody. In this protocol, the concentration of the CF633-conjugated rb anti-dk in the final flow through was too low for fluorescence staining after the two step cross-adsorptions. When this antibody solution was used to stain a specimen already stained with an rb primary antibody and dk anti-rb first secondary antibody, the fluorescent signal was lower than that achieved using the regular staining process. With simultaneous cross-adsorption, CF633-conjugated rb anti-dk was simultaneously cross-adsorbed against the two first secondary antibodies by applying the antibody solution to agarose beads functionalized with the two first secondary antibodies (Fig. S3B). The concentration of the flow through antibody solution containing CF633-conjugated rb anti-dk obtained via simultaneous cross-adsorption had a sufficient concentration for fluorescence staining. This purification process eliminated two types of cross-reaction between the secondary antibodies: [1] the binding of the CF633-conjugated rb anti-dk to the two column-bound secondary antibodies (i in Fig. 1F), and [2] the capturing of the CF633-conjugated rb anti-dk by the two column-bound secondary antibodies (ii in Fig. 1F). When the CF633-conjugated rb anti-dk antibody cross-adsorbed via simultaneous cross-adsorption was used to perform the staining test illustrated in Fig. 1F, no fluorescence signals were imaged in the 633-nm channel (Fig. 1G). This same cross-adsorption process eliminated the crosstalk between the CF568 conjugated ck anti-gt antibody and the rat anti-ms and dk anti-rb antibodies (Fig. S1D to Fig. S1F).

### Elimination of all forms of crosstalk via mutual cross-adsorption

**Figure 3:**
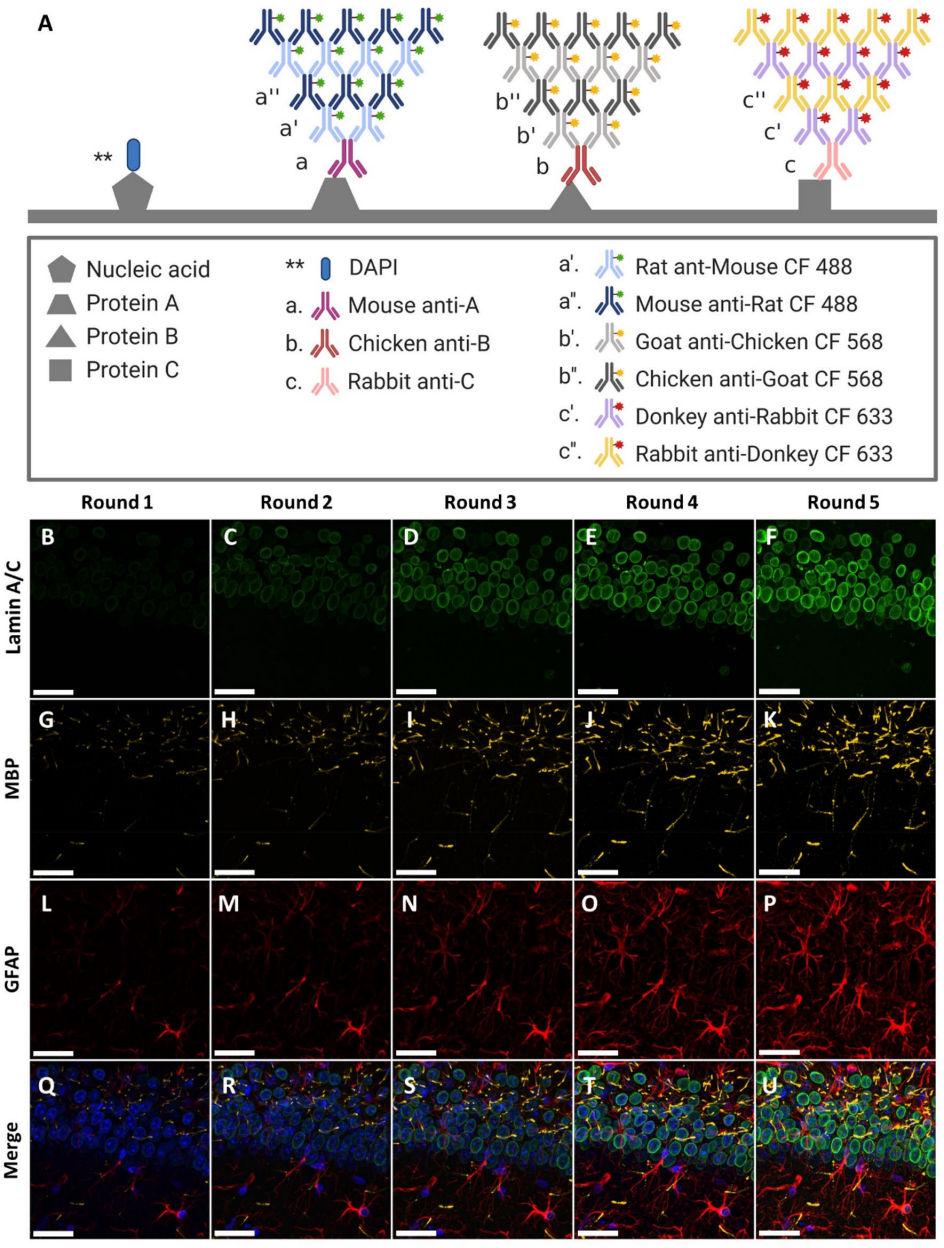
Simultaneous multiplexed IF signal amplification via multiplexed FRACTAL in a mouse brain slice. (**A**) Schematics of the staining process of the multiplexed FRACTAL with imaging results shown in (**B**–**U**). (**B**)–(**U**) Multiplexed IF signal amplification imaging of the CA1 region of a mouse brain. (**B**–**F**) Signal amplification of lamin A/C. (**G**–**K**) Signal amplification of MBP. (**L**–**P**) Signal amplification of GFAP. (**Q**–**U**) Merge images of (**B**–**P**). (**Q**) Merge image of (**B**), (**G**), and (**L**); Round 1. (**R**) Merge image of (**C**), (**H**), and (**M**); Round 2. (**S**) Merge Image of D, I, and N; Round 3. (**T**) Merge Image of (**E**), (**J**), and (**O**); Round 4. (**U**) Merge image of (**F**), (**K**), and (**P**); Round 5. Scale bar: 30 $\mu$m.

**Figure 4:**
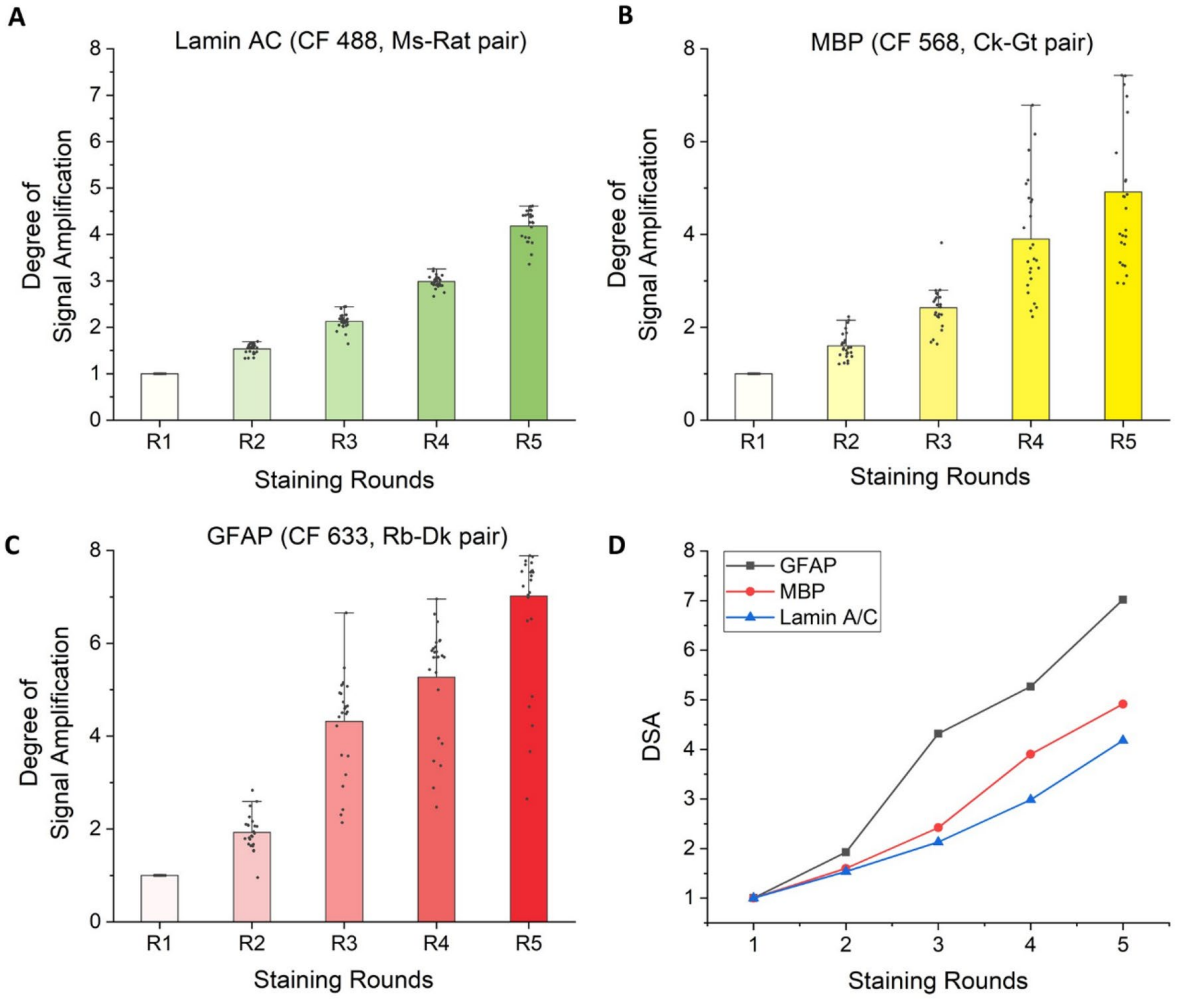
DSA analysis. (**A**–**D**) Degree of signal amplification of each channel: (**A**) lamin A/C. (**B**) MBP. (**C**) GFAP. (**D**) Average degree of signal amplification (DSA) of each channel. The signal intensity was measure at 25 different points during each staining round.

We then performed simultaneous signal amplification of three proteins to investigate whether additional cross-adsorption steps are required for multiplexed FRACTAL. We stained a mouse brain slice with primary antibodies: ms anti-lamin A/C (denoted by a in Fig. 2A), ck anti-MPB (denoted by b in Fig. 2A), and rb anti-GFAP antibodies (denoted by c in Fig. 2A). The brain slice was then stained with the first secondary antibodies: CF488-conjugated rt anti-ms (denoted by a’ in Fig. 2A), CF568-conjugated gt anti-ck (denoted by b’ in Fig. 2A), and CF633-conjugated dk anti-rabbit (denoted by c’ in Fig. 2A). Subsequently, the brain slice was stained with second secondary antibodies: CF488-conjugated ms anti-rt (denoted by a’’ in Fig. 2A), CF568-conjugated ck anti-gt (denoted by b’’ in Fig. 2A), and CF633-conjugated rb anti-dk (denoted by c’’ in Fig. 2A). CF633-conjugated rb anti-dk was used after the cross-adsorption against rt anti-ms and gt anti-ck. As shown in Fig. 2B–E, no crosstalk was observed between the antibodies, as expected. Next, the brain slice was again stained with the three first secondary antibodies: CF488-conjugated rt anti-ms, CF568-conjugated gt anti-ck, and CF633-conjugated dk anti-rb (Fig. 2F–I). However, after staining, GFAP signals appeared in the 488-nm channel, where only the lamin A/C signals should be imaged (Fig. 2F). Such crosstalk may be attributable to one of four crosstalk: [1] binding of rt anti-ms (a’) to dk anti-rb (c’) (denoted by (1) in Fig. 2J), [2] capturing of rt anti-ms (a’) by dk anti-rb (c’) (denoted by (2) in Fig. 2J), [3] binding of rt anti-ms (a’) to rb anti-dk (c’’) (denoted by (3) in Fig. 2K), and [4] capturing of rt anti-ms (a’) by rb anti-dk (c’’) (denoted by (4) in Fig. 2K). We ruled out the first and second crosstalk as the potential cause of the appearance of GFAP signals in the lamin A/C channel, as no GFAP signal was observed in the lamin A/C channel during Round 2 (Fig. 2B). We also expected that the third and fourth crosstalk would not occur, as the cross-adsorption process would have eliminated all forms of potential interaction between these two antibodies. However, the appearance of GFAP signals in the lamin A/C channel during Round 3 indicates that the cross-adsorption we performed did not eliminate all forms of crosstalk.

To eliminate all forms of crosstalk between the antibodies, we performed mutual cross-adsorption between the secondary antibodies. Each secondary antibody was cross-adsorbed against all the orthogonal secondary antibodies. For example, in three-color multiplexed FRACTAL, six secondary antibodies were used, including three first secondary antibodies and three second secondary antibodies. Each secondary antibody was cross-adsorbed against the other four orthogonal secondary antibodies, except its complementary secondary antibody. While performing the cross-adsorption, we again tested serial cross-adsorption and simultaneous cross-adsorption. The concentration of the flow through antibodies from serial cross-adsorption was too low for immunofluorescence staining. To solve this problem, we chose to use simultaneous cross-adsorption. Thus, we functionalized NHS-activated agarose beads in the affinity purification column with all four orthogonal secondary antibodies, applied an antibody solution to the column, and then collected the flow through (Fig. S4). This process was performed for all six secondary antibodies. This mutual cross-adsorption process, which adsorbs one secondary antibody against another and vice versa, eliminated all forms of crosstalk between the secondary antibodies. When these antibodies were used for staining and signal amplification, the fluorescence signals of three proteins: lamin A/C, MBP, and GFAP were simultaneously amplified without any cross-reaction (Fig. 3). We measured the degree of signal amplification (DSA) of the three protein signals, and the DSAs of the three proteins increased linearly with more staining rounds (Fig. 4A–C). These three proteins had different DSAs (Fig. 4D), possibly due to the different degrees to which the secondary antibodies were labeled with fluorescent dyes^17^ or the different binding affinities of the secondary antibodies^18^.

### Multiplexed FRACTAL combined with proExM

**Figure 5:**
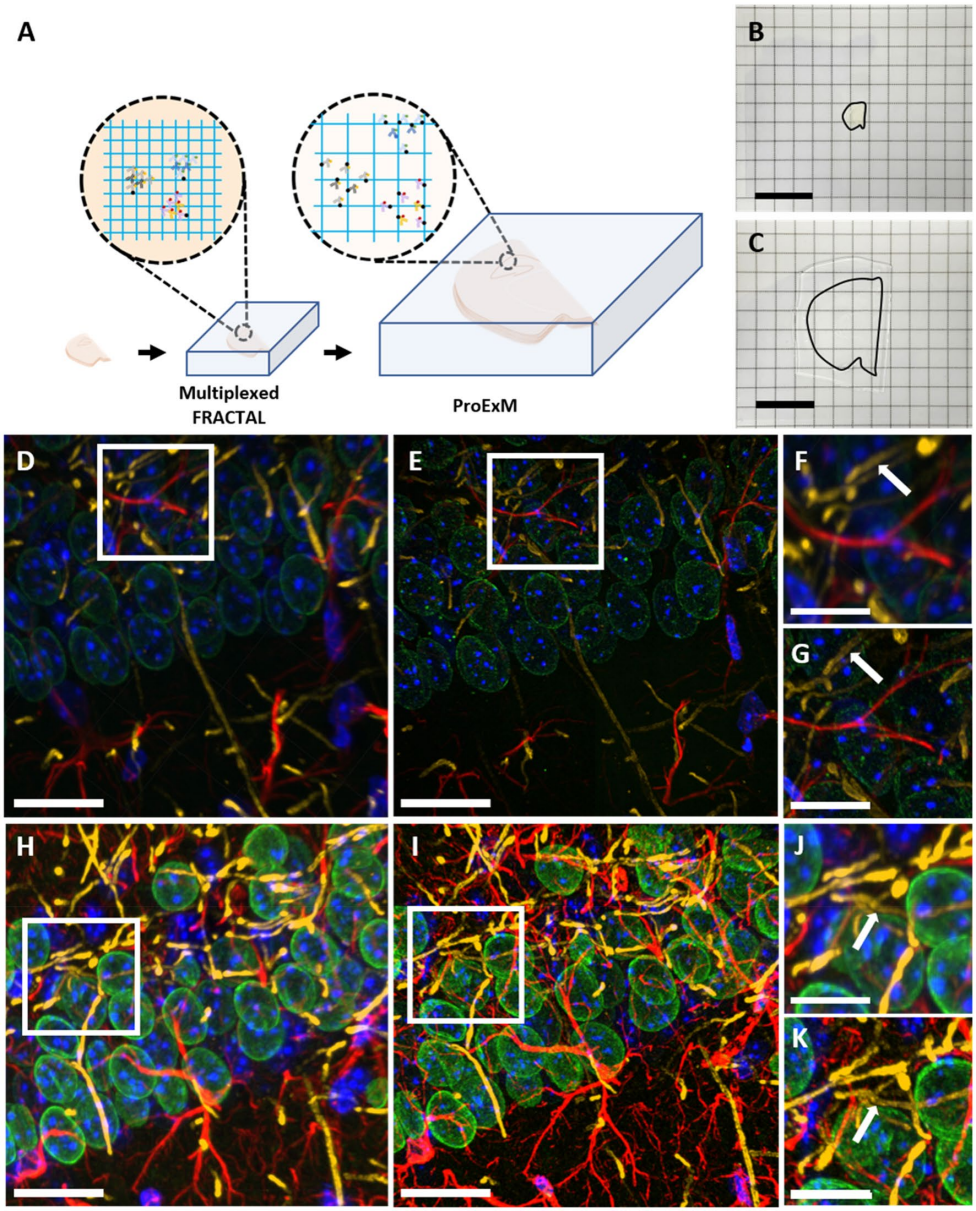
ExM imaging of a multiplexed FRACTAL sample. (**A**) Schematic of multiplexed FRACTAL and ProExM. (**B**) Photograph of a half brain slice before expansion. (**C**) Photograph of the same half brain slice shown in B after expansion. (**D**–**K**) Confocal images of pre-expansion and post-expansion mouse brain samples before and after signal amplification via multiplexed FRACTAL. (**D**–**G**) Before signal amplification: (**D**) Pre-expansion. (**E**) Post-expansion, with expansion factor of 3.30. (**F**) Enlarged image of brain region highlighted in (**D**). (**G**) Enlarged image of highlighted region in (**E**). (**H**–**K**) After six rounds of signal amplification: (**H**) Pre-expansion. (**I**) Post-expansion with expansion factor of 3.16. (**J**) Enlarged image of highlighted region in (**H**). (**K**) Enlarged image of highlighted region in (**I**). (**D**) and (**H**) were obtained under the same acquisition conditions. (**E**) and (**I**) were obtained under the same acquisition conditions. For (**B**) and (**C**), the scale bar is 1.5 cm. For (**D**), (**E**), (**H**), and (**I**), the scale bar is 20 $\mu$m. For (**F**,**G**,**J**), and (**K**), the scale bar is 10 $\mu$m.

Finally, we combined multiplexed FRACTAL with the proExM technique to achieve high signal brightness and super-resolution simultaneously (Fig. 5A–C). One of the problems of expansion microscopy (ExM) is the low intensities of the fluorescence signals after expansion. Most fluorophores lose around 50% of their fluorescence during the free-radical polymerization process^19^ and their fluorescence intensities decrease further due to volumetric dilution of the fluorophores after expansion. We investigated whether multiplexed FRACTAL is compatible with ExM for achieving higher fluorescence intensities after expansion. A 150 μm thick mouse brain slice was first stained with antibodies against lamin A/C, MBP, and GFAP and their signals were simultaneously amplified via cyclic staining with the six secondary antibodies that were cross-adsorbed. A separate mouse brain slice was stained using regular indirect antibody staining without any signal amplification. When these two slices were imaged with the same imaging conditions, the difference in their signal intensities was apparent (Fig. 5D,H). These two brain slices were then expanded via proExM protocol and their fluorescence signal intensities were compared. When the two expanded specimens were imaged with the same imaging conditions, the difference in their signal intensity was again apparent (Fig. 5E,I). In both specimens, detailed nanoscale structures that were not visible before expansion were clearly imaged after expansion. For example, the hollow tubular structures of MBP that were not visible before expansion (white arrow in Fig. 5F) were resolved only after expansion (white arrow in Fig. 5G) in a non-amplified specimen. Similarly, in the amplified specimen, the MBP structures that were blurry before expansion (Fig. 5J) were also resolved in the amplified specimen (white arrow in Fig. 5K). Both before and after expansion, the fluorescence signal intensities of the FRACTAL-processed specimen were five times higher than that of the non-processed specimen, after five rounds of signal amplification. The imaging depth before the expansion was determined by the length of the antibody staining. In this work, the mouse brain slices were stained with both primary and secondary antibodies for 10 h, and the antibodies were diffused up to 20 μm from the brains’ surface. We believe that extending the staining time would have resulted in a deeper staining depth. In our experiments, the mouse brains expanded 3.16-fold; the thickness of the slices was 150 μm before expansion and 474 μm after expansion.

## Discussion

In this study, we show that simultaneous signal amplification of multiple fluorescence signals can be achieved via FRACTAL with secondary antibodies that are mutually cross-adsorbed. By using commercial affinity purification columns and simultaneous cross-adsorption, cross-reactivity between multiple secondary antibodies were eliminated through a simple cross-adsorption process. Using these secondary antibodies, the fluorescence signal intensities of three proteins were simultaneously amplified by approximately 4 to sevenfold after five rounds of staining. Finally, we show that the multiplexed FRACTAL is compatible with a tissue expansion technique.

Recently, various multiplexed imaging techniques have been developed to meet the increasing need for simultaneous imaging of multiple proteins from a single specimen^20–22^. FRACTAL can be combined with such multiplexed imaging techniques to achieve multiplexed imaging of proteins with low expression levels. In this study, we demonstrate simultaneous immunofluorescence signal amplification of three proteins. However, considering the host of commercially available secondary antibodies, up to of four or five signals can be amplified simultaneously. Potentially, simultaneous signal amplification of more proteins can be achieved using fragment crystallizable (Fc) engineered antibodies derived from microbial hosts such as E. coli^23^. The Fc region of secondary antibodies can be easily modified or functionalized with other proteins such as protein A, protein G, and protein Z^24^. When such antibodies produced from microorganisms are used, only one host would be required to amplify a single protein signal. For example, a mouse brain slice stained with ms anti-GFAP—a microbial antibody—against ms bearing protein G at its Fc region, and then ms anti-protein G. By applying the microbial antibody and ms anti-protein G multiple times, the signal of the GFAP can be amplified. To perform multiplexed signal amplification using this approach, the mutual cross-adsorption process we demonstrate in this study would be crucial for eliminating any potential cross-reactivity between antibodies with a single process.

Multiplexed FRACTAL is a simple but highly versatile signal amplification technique that can be performed easily in standard biology laboratories because it utilizes commercial antibodies and affinity purification columns. This technique can be employed in various applications that require the detection of multiple targets with high sensitivity, such as drug screening and drug development^25^. Furthermore, it can be applied in the pathological examination of tissue slices and in cancer diagnostics, which require highly-sensitive detection of multiple proteins via microscope images^26^. FRACTAL is also expected to work with immunostaining-compatible cleaning techniques that do not use hydrogels. To show that FRACTAL is compatible with such clearing techniques, we applied it to a mouse brain slice and cleared it using BABB^27^. We found that the FRACTAL signals were maintained after the clearing. We added this result as Figure S5. The compatibility of FRACTAL with hydrogel-based clearing techniques, such as clear, lipid-exchanged, acrylamide-hybridized rigid, imaging/immunostaining compatible, tissue hydrogel (CLARITY), should be further studied^28^.

## Methods

All the chemicals and reagents used in this work are listed in Supplementary Table 1.

### Mouse brain Perfusion and Sectioning

All of the animal experiments in this article were carried out in accordance with relevant guidelines and regulations that were approved by the Korea Advanced Institute of Science and Technology Institutional Animal Care and Use Committee (KAIST-IACUC). All of the animal experiments in this study were carried out in compliance with the ARRIVE (Animal Research : Reporting of In Vivo Experiments) guidelines. C57BL/6 J mice (Aged 8–14 weeks) that were grown at specific pathogen free (SPF) facilities were anesthetized with isoflurane and transcardially perfused with 4% paraformaldehyde (PFA) in 1 × phosphate-buffered saline (PBS). The brains were then further fixed with 4% PFA in 1 × PBS for 2 h at 4 ℃. The brains were sectioned with a vibratome (Leica VT1000S) to thickness of 150 $\upmu$m and stored in a storage buffer (0.1 M glycine, 0.01% sodium azide in 1 × PBS) at 4 ℃ until needed. From the literature, we discovered postfixation lengths ranging from 0 to overnight. We experimented with several lengths and found that 2 h produced the best staining results. The same length was also used in^29^.

### Secondary antibody-fluorophore conjugations

First, 90 μL of each antibody solution with a concentration of 1 mg/ml was mixed with 10 μL of 1 M sodium bicarbonate buffer (1 M NaHCO_3_, pH 8.3). And then, NHS-ester fluorophore in dimethyl sulfoxide (DMSO) was added to the mixture. The ratio of secondary antibody to fluorophore was set to be 9:1 for optimal conjugation. The solution was kept at room temperature for 1 h. The fluorophore-conjugated antibodies were purified from the unconjugated antibodies or residual fluorophores through NAP-5 gel filtration columns. The NAP-5 gel filtration columns were used after equilibration by flowing the sufficient amount of 1 × PBS according to manufacturer’s instructions. The fluorophore-antibody solution was added to the columns with 400 μL of 1 × PBS. After two fluorescent bands were separated, fluorophore-conjugated antibodies that were located at the bottom band were collected after flowing 500 μL of 1 × PBS. The collected antibody solution was purified again and concentrated using 30 K centrifugal filters with centrifugation (14,000 g, 10 min, 4 ℃). Finally, 1 × PBS was added to the concentrated antibody solution up to 90 μL of its total volume and stored at 4 ℃ until needed.

### Affinity purification against orthogonal secondary antibody

For antibody affinity purification process, NHS-activated agarose gel spin column was used. First, 50 μL of each orthogonal secondary antibody solution (for example, ms anti-rt, rt anti-ms, chk anti-gt and gt anti-ck secondary antibodies) were added to the column with 200 μL of 1 × PBS, simultaneously. The solution was incubated for 2 h at room temperature or overnight at 4 ℃. After washing with 1 × PBS, quenching solution (1 M Tris, pH 7.4) was added to the column and kept at room temperature for 15 min. After another washing with 1 × PBS, 50 μL of antibody solution which should be purified was added to the column with 350 μL of 1 × PBS and left for 2 h at room temperature or overnight at 4 ℃. Finally, flow-through antibody solution was collected and concentrated using 30 K centrifugal filters with centrifugation (14,000 g, 10 min, 4 ℃). Then 1 × PBS was added up to 40 μL of its total volume and stored at 4 ℃ until needed.

### Multiplexed FRACTAL immunostaining of mouse brain slices

All of the following steps were performed at room temperature. For all of the following staining procedures, a primary antibody was diluted to 2.5 μg/mL in MAXbind™ Staining Medium and secondary antibodies were diluted to 5.2 μg/mL in MAXbind™ Staining Medium. All washing steps were performed using MAXwash™ Washing Medium. 150 μm thick mouse brain slice was blocked and permeabilized with MAXblock™ Blocking Medium for 2 h. For the primary antibody staining, the slice was stained with mouse (ms) anti-rat (rt), chicken (ck) anti-goat (gt) and rabbit (rb) anti-donkey (dk) primary antibodies for 4 h and then washed three times. For the first secondary antibody staining, the sample was stained with CF 488 conjugated rt anti-ms, CF 568 conjugated gt anti-ck and CF 633 conjugated dk anti-rb secondary antibodies for 4 h and washed three times. For the second secondary antibody staining, the sample was stained with CF 488 conjugated ms anti-rt, CF 568 conjugated ck anti-gt and CF 633 conjugated rb anti-dk secondary antibodies for 4 h and washed three times. These staining procedures were repeated several times. All of the washing steps were carried out for 20 min.

### Protein-retention expansion microscopy with pre-gel staining (pro-ExM)

The stained mouse brain slices were treated with 0.1 mg/mL of acryloyl-X, SE (6-((acryloyl)amino) hexanoic acid, succinimidyl ester (AcX) in 1 × PBS for overnight at room temperature, and washed three times with 1 × PBS for 30 min. The brain slices were incubated in a gelation solution (7.5% (w/w) sodium acrylate, 2.5% (w/w) acrylamide, 0.15% (w/w) N,N’-methylenebisacrylamide, 2 M sodium chloride (NaCl), 0.2% (w/w) ammonium persulfate (APS), 0.2% (w/w) tetramethylethylenediamine (TEMED), and 0.01% (w/w) 4-hydroxy-2,2,6,6-tetramethylpiperidin-1-oxyl (H-TEMPO) in 1 × PBS) for 30 min at 4 ℃ and 1.5 h at 37 ℃. After gelation, the brains were incubated in the digestion buffer (1 mM ethylenediaminetetaacetic acid (EDTA), proteinase K diluted in the ratio of 1:100 in 1 × PBS) overnight at room temperature. After digestion, the gels were immersed in DI water, and DI water was exchanged every 15 min for 4 times with gentle shaking.

### Imaging with FM

Images shown in Figs. 1C–E,G, 2B–I, 3B–U and 5D–K were obtained with an Andor spinning disk confocal microscope with a 40 × 1.15 NA water immersion objective. Detailed imaging acquisition conditions were shown in Supplementary Table 2.

### Determination of the degree of signal amplification

To determine the degree of signal amplification (DSA) of multiplexed FRACTAL processed samples shown in Fig. 3B–U, the images were acquired at same region in dentate gyrus of the mouse brain for each staining round. The max intensity measurement was performed using the ImageJ image-processing program. Line intensity profiles perpendicular to each protein structure (lamin A/C, MBP and GFAP) were measured at 25 different points, and it was repeated every staining round at the same location.

## Supplementary Information

Supplementary Information.
