# Supplementary material for: Simultaneous amplification of multiple immunofluorescence signals via cyclic staining of target molecules using mutually cross-adsorbed antibodies
Source: Sci Rep. 2022 May 24;12:8780. doi: 10.1038/s41598-022-12808-y (PMC9130514; doi:10.1038/s41598-022-12808-y)
Supplement: Supplementary file 1 — Supplementary Information. [file 41598_2022_12808_MOESM1_ESM.docx]

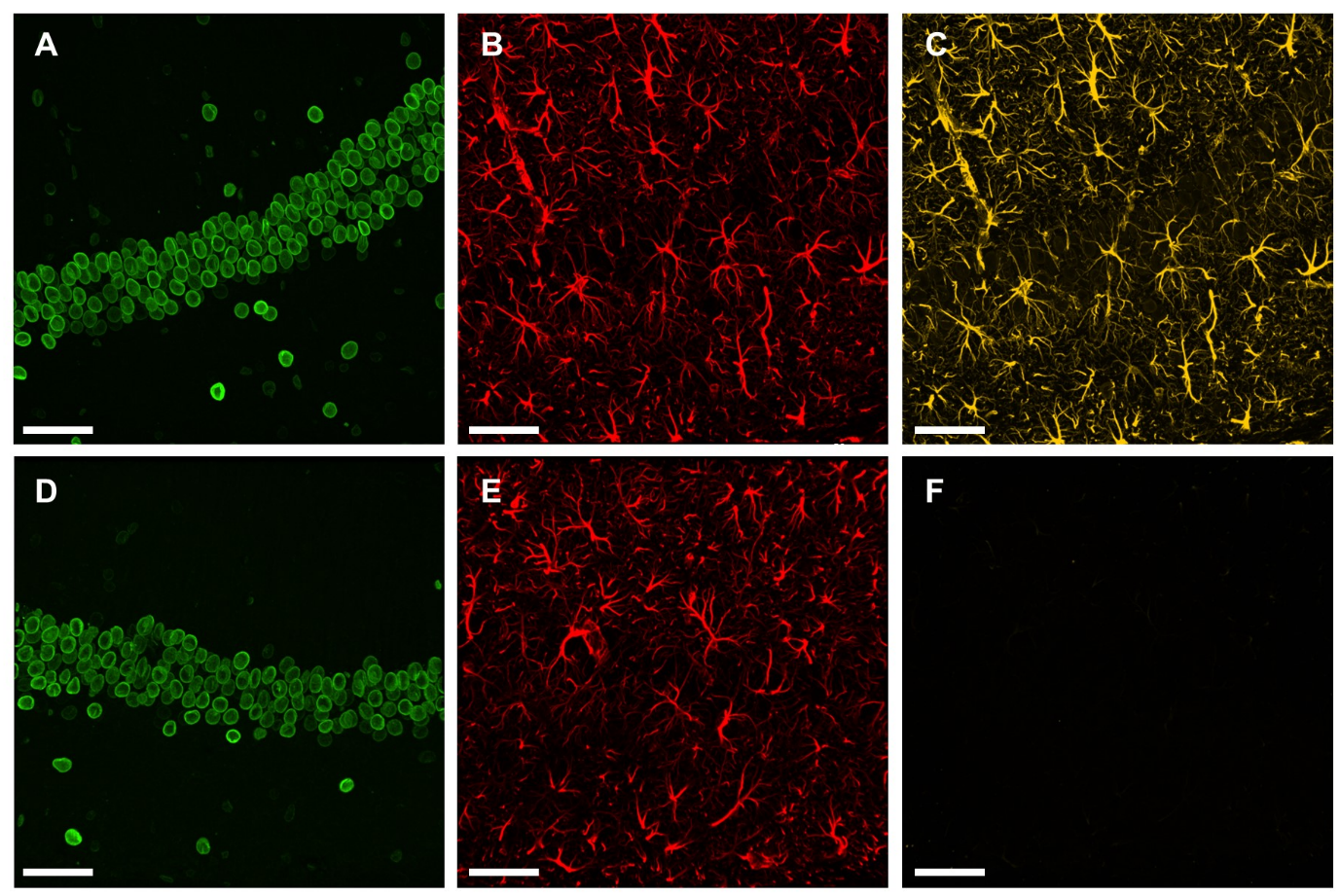


Fig S1. Purification of ck anti-gt secondary antibody

(A)-(F) Confocal images of lamin A/C, GFAP and MBP structures at CA1 region of the mouse brain slice. (A) 488-nm channel : lamin A/C. (B) 633-nm channel : GFAP. (C) 568-nm channel, using unpurified ck anti-gt secondary antibody; overlaps with 488 and 633-nm channels. (D) 488-nm channel : lamin A/C. (E) 633-nm channel : GFAP. (F) 568-nm channel : using purified ck anti-gt secondary antibody; no signal was observed. For (A)-(F), scale bars are 30 $\mu$m.

**
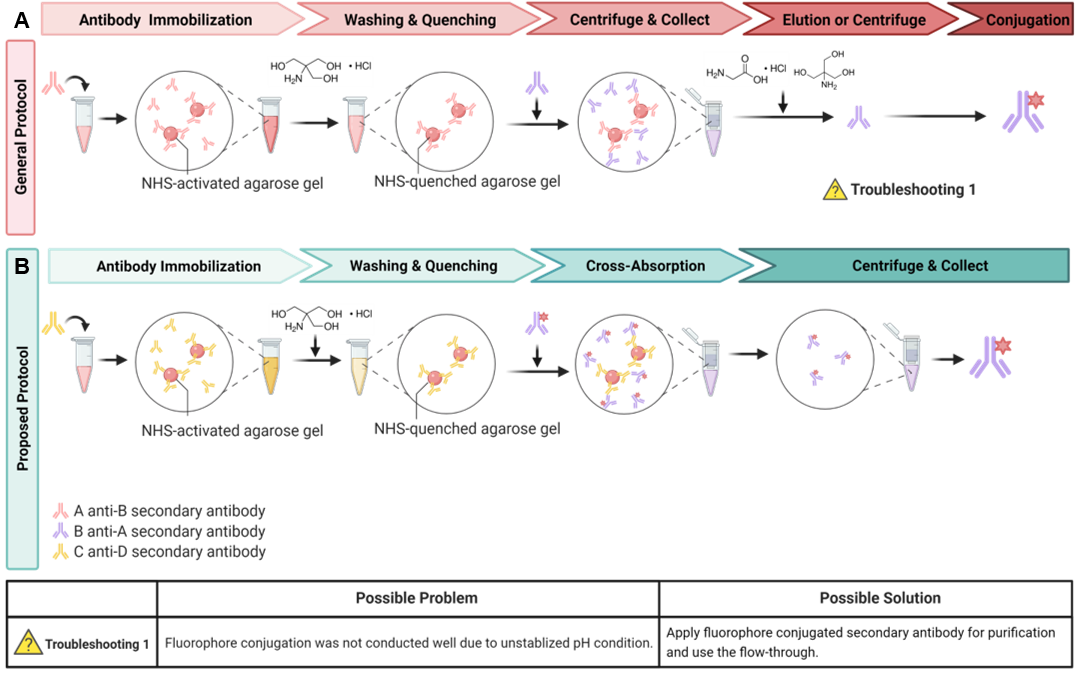
**

**Fig S2. General suggested protocol of NHS-activated agarose gel and modified antibody purification protocol**

(A) Commercial method of using affinity purification column, which aims at collection of antibodies with a strong affinity against specific target. (B) Modified method of using affinity purification column for collecting antibodies with no afiinity against specific targets.

**
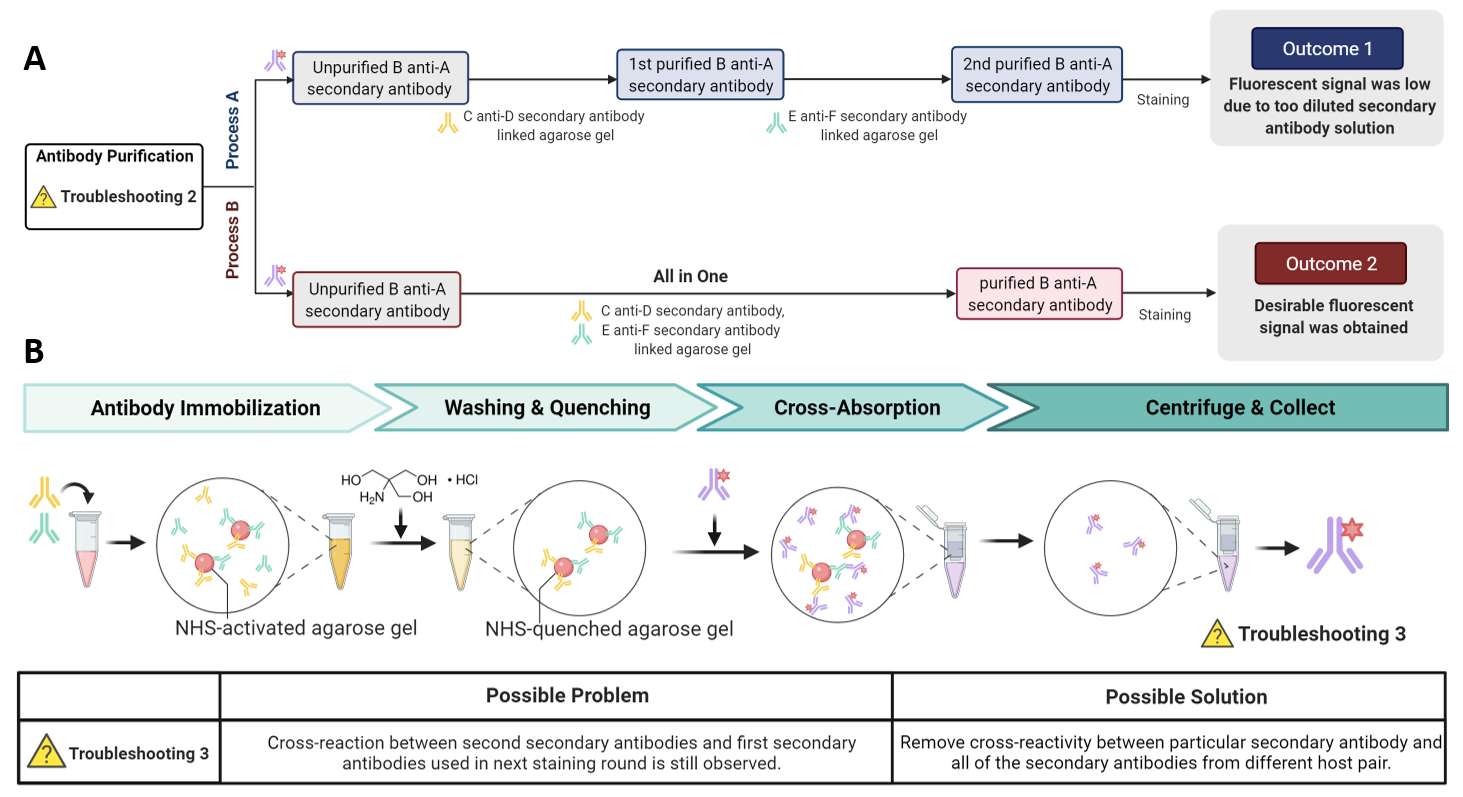
**

Fig S3. Antibody purification against two orthogonal secondary antibodies

(A) Process A : Serial purificatin of secondary antibody against two orthogonal secondary antibodies, which results in low concentration of antibody. Process B : Simultaneous purification of secondary antibody against two orthogonal secondary antibodies. (B) Schematics of purification method, Process B. Purified second secondary antibodies through this method still cross-reacts with first secondary antibody used in next staining round.


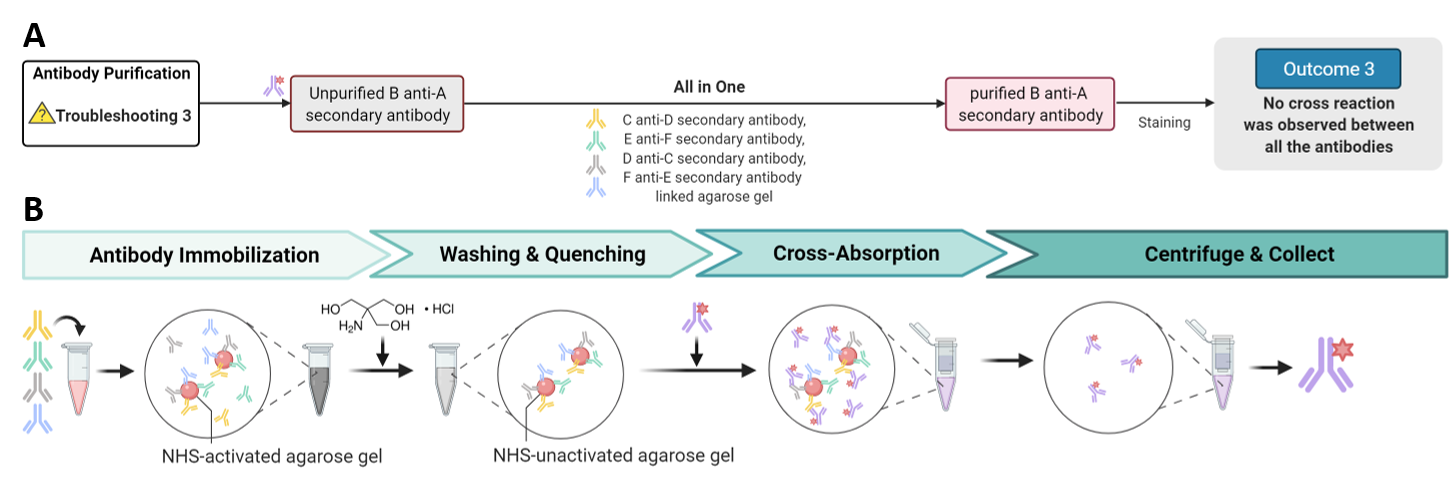


Fig S4. Mutual antibody purification against all of the orthogonal secondary antibodies

(A) Simultaneous purification of secondary antibody against four orthogonal secondary antibodies. It results in no observed cross-reaction between all of the used antibodies. (B) Schematics of mutual antibody purification. It provides proper way to conduct multiplexed FRACTAL without any cross-reaction.


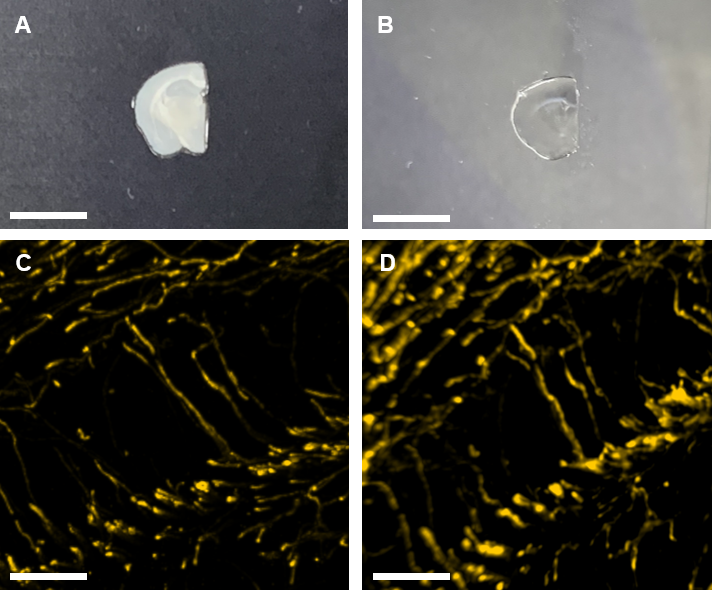


Fig S5. FRACTAL with BABB clearing

(A) Brain slice, before BABB clearing. (B) Brain slice, after BABB clearing. (C)-(D) Confocal images of MBP, 561-nm channel. (C) Round 1, without BABB clearing. (D) Round 3, after BABB clearing. For (A) and (B), the scale bar is 6 mm. For (C), the scale bar is 30 $\mu$m. For (D), the scale bar is 25 $\mu$m.

| **Product name** | **Vendor** | **Product number** |
| --- | --- | --- |
| **Mouse brain fixation** | | |
| 16% paraformaldehyde (PFA) | Electron Microscopy Science | 15710 |
| Glycine | Sigma | 50046 |
| Sodium azide | Sigma | 71289 |
| Triton X-100 | Sigma | X100 |
| 10× PBS | Invitrogen | AM9625 |
| **Staining Solution** | | |
| MAXblock™ Blocking Medium | Active Motif | 15252 |
| MAXbind™ Staining Medium | Active Motif | 15251 |
| MAXwash™ Washing Medium | Active Motif | 15254 |
| **Antibody** | | |
| Mouse anti-lamin A/C antibody | SYSY | 4777S |
| Chicken anti-MBP antibody | Aves labs | MBP |
| Rabbit anti-GFAP antibody | ATLAS | HPA056030 |
| AffiniPure Donkey Anti-Rabbit IgG (H+L) | JacksonImmunoResearch | 711-005-152 |
| Rabbit anti-Donkey IgG (H+L) Secondary Antibody | Thermo Fisher | SA1-26816 |
| AffiniPure Goat Anti-Chicken IgY (IgG) (H+L) | JacksonImmunoResearch | 103-005-155 |
| Chicken anti-Goat IgG (H+L) Secondary Antibody | Novus biologicals | NBP1-74804 |
| AffiniPure Mouse Anti-Rat IgG (H+L) | JacksonImmunoResearch | 212-005-168 |
| AffiniPure Rat Anti-Mouse IgG (H+L) | JacksonImmunoResearch | 415-005-166 |
| **Fluorophore** | | |
| CF 488 | Biotium | 92120 |
| CF 568 | Biotium | 92131 |
| CF 633 | Biotium | 92133 |
| DAPI | Sigma | D9542 |
| **Fluorophore conjugation** | | |
| Sodium bicarbonate | Sigma | S6297 |
| NAP-5 columns | Cytiva | 17-0853-02 |
| Amicon^®^ Ultra-0.5, 30K MWCO | Merck Millipore | Z740174 |
| **Antibody purification** | | |
| Pierce NHS-Activated Agarose Spin Columns, 0.2 mL | ThermoFisher | 26198 |
| Trizma hydrochloride (Tris-HCl), pH7.4, 1 M | Sigma | T2194 |
| **Expansion microscopy** | | |
| Sodium acrylate | Sigma | 408220 |
| Acrylamide | Sigma | A9099 |
| N, N'-methylenebisacrylamide (BIS) | Sigma | M7279 |
| 4-hydroxy-TEMPO (H-TEMPO) | Sigma | 176141 |
| Ammonium persulfate (APS) | Sigma | A3678 |
| N,N,N',N'-tetramethylethylenediamide (TEMED) | Sigma | T7024 |
| Acryloyl-X SE (AcX) | Thermofisher | A-20770 |
| Proteinase K | New England Biolabs | P8107S |
| Ethylenediaminetetraacetic acid (EDTA) | Sigma | EDS |
| Sodium chloride | Sigma | 71376 |
| Triton X-100 | Sigma | X100 |

Table S1. List of materials used in this study.

**
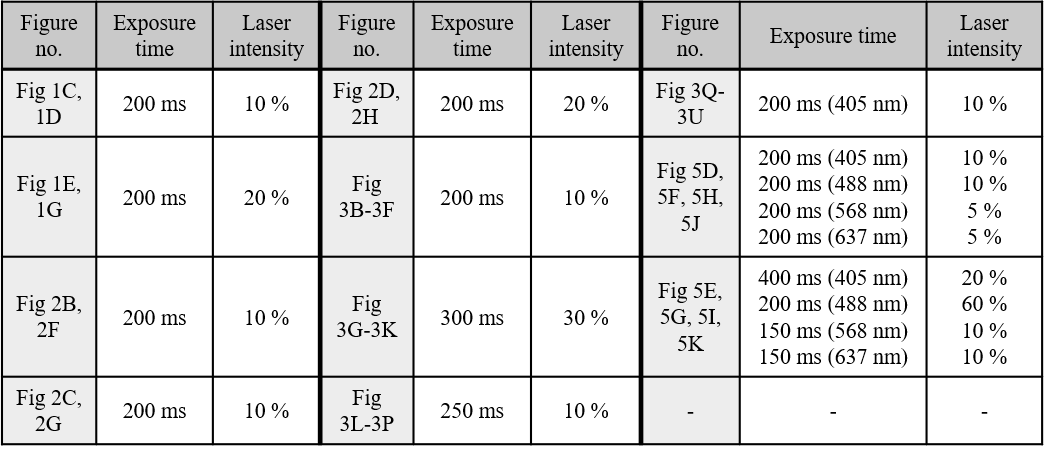
**

Table S2. Imaging acquisition conditions of each figure.
